# Diagnostic dilemma in the management of a cervical spinal cord lesion: neuromyelitis optica spectrum disorder: a case report

**DOI:** 10.1186/s13256-026-06420-1

**Published:** 2026-08-14

**Authors:** Kelechi Michael Azode, Simon Adewale Balogun, Morenikeji Adeyoyin Komolafe, Babatunde Josiah Olasode, Edward Oluwole Komolafe

**Affiliations:** 1 Neurosurgery Unit, Department of Surgery, Obafemi Awolowo University Teaching Hospitals Complex, Ile-Ife, Osun Nigeria; 2 Department of Medicine, Obafemi Awolowo University Teaching Hospitals Complex, Ile-Ife, Osun Nigeria; 3 Department of Morbid Anatomy and Forensic Medicine, Obafemi Awolowo University Teaching Hospitals Complex, Ile-Ife, Osun Nigeria

**Keywords:** Neuromyelitis optica, Cervical spine lesion, Aquaporin, Transverse myelitis

## Abstract

**Background:**

Neuromyelitis optica spectrum disorder, known eponymously as Devic’s disease, is a condition in which antibodies are elaborated against aquaporin water channels in the end foot processes of astrocytes due to autoimmune dysregulation thereby initiating a cascade of neuroinflammatory events that culminates in astrocytic injury, dysfunctional axonal blebs and demyelination of the central nervous system neurons. Its cervical spine symptoms may sometimes mimic a similar presentation of a cervical spine lesion. This is further validated by its core clinical magnetic resonance image feature of longitudinally extensive transverse myelitis bearing radiologic similarities with an intramedullary spinal cord lesion thereby resonating the need to maintain a high index of suspicion when evaluating patients with either condition. It is pertinent to highlight that while surgery may confer a potential benefit in patients with acute exacerbations of symptoms attributable to cervical spine compressive myelopathy due to the disease, the mainstay of treatment involves the use of steroids, immunosuppressive therapy and plasmapharesis.

**Case presentation:**

We report a case of a 50-year-old Nigerian female of Yoruba ethnicity who presented with progressive weakness involving all limbs of 2 weeks’ duration. She had associated history of photophobia and a 1-week history of fever with sore throat a month prior to presentation. Cranial and cervical spine T2-weighted magnetic resonance imaging showed increase signal intensity within the right optic nerve and bulbous expansion with signal intensity noted in the C3–C7 segments of the spinal cord, respectively. She had C3–C6 cervical decompression surgery to relieve her symptom of progressive weakness of all her limbs with associated worsening difficulty with breathing. Laboratory investigations parameters were in keeping with neuromyelitis optica spectrum disorder and histological analysis of resected tissue specimen revealed features of neuroinflammation. She was placed among other medications, prednisolone, tenofovir, and azathioprine. She died on postoperative day 27 following development of sudden-onset respiratory distress.

**Conclusion:**

This article highlights the need to suspect a diagnosis of Devic’s disease when evaluating patients with cervical spine lesions. It underscores the need for early diagnosis, the need for intensive ventilatory support, and the potential role of immunosuppressive therapy in improving outcomes.

## Introduction

Neuromyelitis optica spectrum disorder is a disease condition of the central nervous system in which there is an autoimmune complement and antibody-mediated cellular destruction causing injury to the astrocytes with an impediment to their functions such as the perpetuation of immune responses, propagation of synaptic transmission, and maintenance of the integrity of the blood–brain barrier [1].

Described by Eugene Devic in 1894, the disease condition, which was previously attributed to bear similarity with multiple sclerosis, has been distinguished from the former as an autoimmune astrocytopathy following the discovery of antibodies IgG that bind to the water channel aquaporin-4 located in the astrocytic end foot process. This stimulates a cascade of cell-mediated and antibody dependent destruction of astrocytes with consequent loss of oligodendrocytes leading to demyelination [2].

Given an annual worldwide prevalence range of 0.5 to 4 per 100,000, the highest incidence and prevalence rate of 0.73 per 100,000 and 10 per 100,000 persons, respectively, have been documented in the Afro-Caribbean regions with a 40-year mean age of onset and a male-to-female ratio of 1:9 [3]. A systematic review of eligible studies in Africa documented 410 cases reported in 10 African countries mainly domiciled in the North Africa subregion with a median age of diagnosis of 33 years and female preponderance of 75% [4].

While documented reports have shown patients with spastic quadriplegia, tonic spasms, neck pain with a positive Lhermitte sign, and biochemical features of antiphospholipid syndrome, [5] the core clinical features of the disease condition include longitudinally extensive transverse myelitis, optic neuritis, area postrema syndrome, and brainstem syndromes with presentation of gaze palsies, hearing loss, and difficulty with breathing and swallowing, which are indicative of cranial nerve involvement [6].

Given the rarity of the disease condition, its clinical manifestation of symptoms that may be suggestive of other common prevalent conditions such as a high cervical spine lesion and the relative unavailability and high cost of diagnostic modalities in a resource-limited setting, its clinical presentation often presents a diagnostic dilemma which requires a high index of suspicion and clinical acumen to diagnose.

## Case presentation

A 50-year-old right-handed Nigerian female of Yoruba ethnicity presented to us with progressive weakness involving all limbs of 2 weeks’ duration. The weakness started from the left shoulder, then the left upper limb and progressed in a cyclical pattern until the patient became quadriplegic over a 2-week duration. There was a history of a 1-week fever and sore throat which occurred a month before presentation and resolved when she took antibiotics and antimalaria medication. There was a positive history of photophobia with no blurring of vision or diplopia. She had recurrent episodes of vomiting with no other features suggestive of raised intracranial pressure. There was difficulty with swallowing, however there was no hearing loss nor dysphagia. She had no history of similar symptoms in a first-degree family relative.

Pertinent findings on examination were essentially in the neurologic system where she had an increased tone and deep tendon reflexes (Table 1) with motor and sensory levels bilaterally at C4 myotome and C5 dermatomes, respectively. Joint position and vibration sense were absent bilaterally. Per rectal examination showed an absent perianal and deep anal pressure sensation, a lax anal sphincteric tone and an absent bulbocavernosus reflex. She had features of lower cranial nerve palsies such as difficulty with deglutition and weakness of the tongue with deviation to the left.

**Table 1:** Musculoskeletal examination findings

Motor examination	Right upper limb	Right lower limb	Left upper limb	Left lower limb
Bulk	Normal	Normal	Normal	Normal
Tone	Increased	Increased	Increased	Increased
Power	0	0	0	0
Reflexes	Increased	Increased	Increased	Increased

We entertained a diagnosis of a high cervical spinal cord tumor with the possibility of a foramen magnum lesion and demyelinating diseases of the central nervous system such as multiple sclerosis, neuromyelitis optica spectrum disorder, and myelin oligodendrocyte associated glycoprotein disorder. She had laboratory blood investigations, the results of which are shown in Table 2.

**Table 2:** Laboratory investigation results

Parameter	Value	Interpretation
Packed cell volume	45%	Normal
White blood cell count	7000 cells/mm^3^	Normal
Platelets	213,000 cells/mm^3^	Normal
Creatinine	151 mmol/L (50–132 mmol/L)	Elevated
Urea	12.5 mmol/L (2.5–5.8 mmol/L)	Elevated
Potassium	3.5 mmol/L (3–5 mmol/L)	Normal
Sodium	135 mmol/L (120–140 mmol/L)	Normal
Bicarbonate	22 mmol/L (20–30 mmol/L)	Normal
Hepatitis B surface antigen	Positive	

**Fig. 1:**
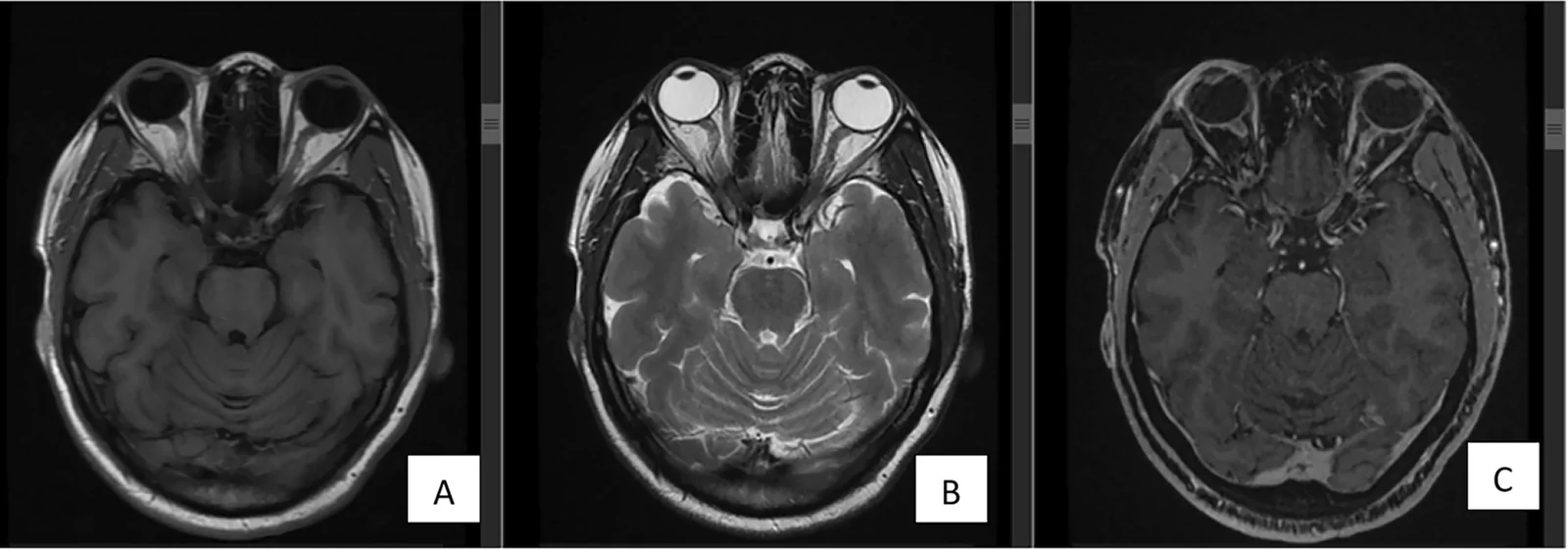
Brain magnetic resonance imaging scan showing hyperintensity in the course of the right optic nerve on T1-weighted image (**A**), with a relatively increased signal intensity within the nerve compared to the left on T2-weighted image (**B**) and decreased contrast uptake in the right optic nerve (**C**)

A brain magnetic resonance image showed an impingement in the course of the right optic nerve with decreased T2 signal intensity within the nerve close to the optic chiasm and decreased contrast uptake (Fig. 1).

**Fig. 2:**
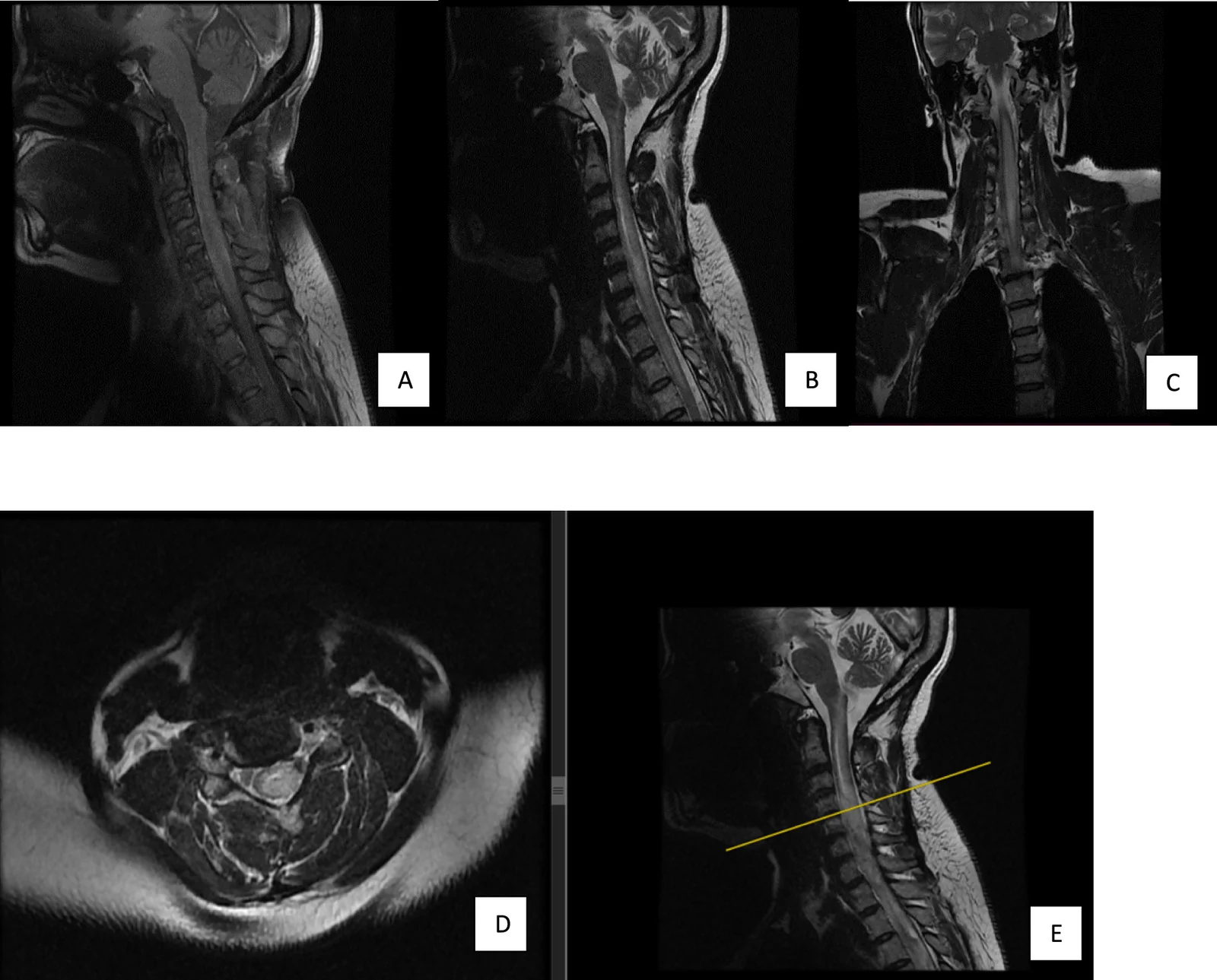
Cervical spine magnetic resonance imaging scan with (**A**) Sagittal T1 image showing isointense bulbous expansion of the cord from C3–C7 spinal segments. (**B**) and (**C**) Sagittal and coronal T2-weighted images showing increased signal intensity within the bulbous spinal cord expansion extending from C3–C7 spinal segments. **D** T2-weighted image showing an axial representation of a sagittal slice (**E**) of the spinal cord at C4/C5 segment showing the lesion to be intramedullary

A cervical spine MRI scan revealed a contrast-enhancing longitudinal intramedullary spinal cord lesion extending from C3 to T2 spinal segment with bulbous expansion of the cord, increased signal on T2-weighted image and decreased CSF signal on myelogram of the involved spinal cord segments (Fig. 2).

On the 3rd-day postadmission, she developed severe respiratory distress thereby necessitating admission into the intensive care unit. This also informed the decision for emergency surgical exploration with the aim of spinal cord decompression to create space for the already-expanded cord within the cervical vertebral canal and relieving the possible pressure transmitted from the cervical spinal cord to the phrenic nerves that innervate the diaphragm for its respiratory function, while serum samples for aquaporin-4 IgG antibodies and oligoclonal bands were sent to the laboratory for assay to rule out other differential diagnosis.

**Fig. 3:**
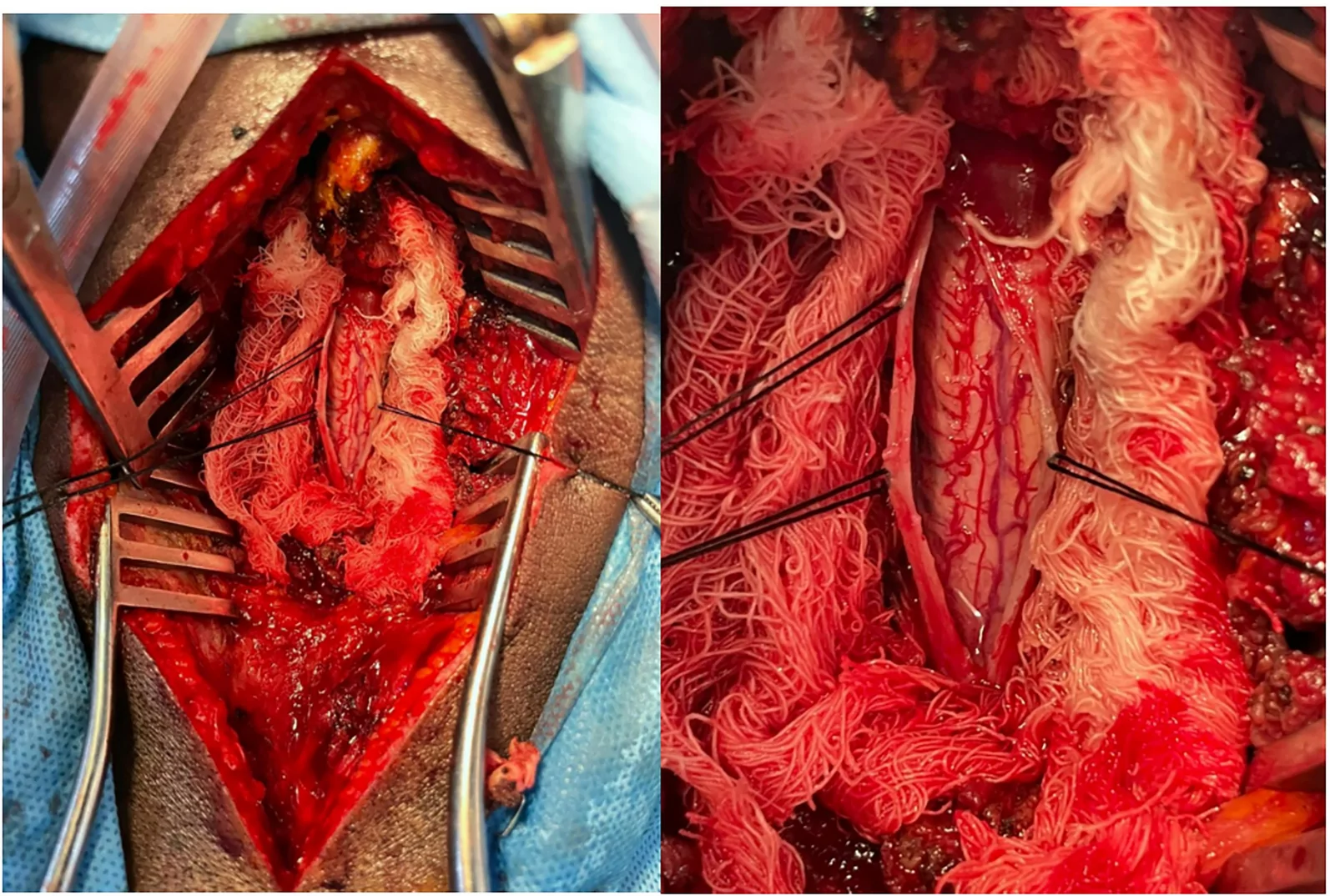
Intraoperative pictures showing a normal spinal cord appearance with no extramedullary nor intramedullary lesion visible. Black-colored silk sutures are used to tag and reflect the dura mater on both sides, thereby exposing the cervical spinal cord with its adherent posterior spinal vessels

On the 4th day of admission, due to diagnostic uncertainty coupled with progressively worsening respiratory distress, deteriorating clinical condition with a potential imminent risk of mortality, she had a C3–C6 Laminectomy with intramedullary exploration and biopsy of a C5 spinal cord segment for histologic analysis. Intraoperative findings were a normal-looking dura and normal spinal cord consistency with no obvious intramedullary tumor (Fig. 3).

Due to the relative rarity of neuromyelitis optica spectrum disorder in our environment and the consequent unavailability of laboratory reagents in our hospital to analyze for aquaporin-4 IgG antibodies, upon admission her blood samples were sent to another facility where the resources for the analysis were available. On the 2nd day postop, her laboratory results returned with elevated titers of aquaporin-4 IgG antibodies (1:32), while serum myelin oligodendrocyte glycoprotein antibody titers were not elevated. Her D-dimer and C-reactive protein levels were elevated, while her other investigation results were within the normal range of values (Table 3).

**Table 3:** Laboratory investigations showing elevated titers of serum AQP4 antibody and other acute-phase proteins

Parameter	Value	Interpretation
Serum AQP4 antibody	Titer 1:32	Elevated
Serum myelin oligodendrocyte antibody	Titer 1: < 10	Not elevated
D-dimer	> 10 (0–0.5 mg/L)	Elevated
Serum lactate	0.87 (0.5 −2.2 mmol/L)	Normal
C-reactive protein	255.33 (< 10 mmol/L)	Elevated

Postoperatively she was managed in the intensive care unit on ventilatory support through an endotracheal tube, had nasogastric tube feeding, and was placed on meropenem, metronidazole, LMW heparin, azathioprine, prednisolone, rabeprazole, hematinic, tenofovir, vitamin D, and warfarin. She was also commenced on cardiopulmonary and neurophysiotherapy.

**Fig. 4:**
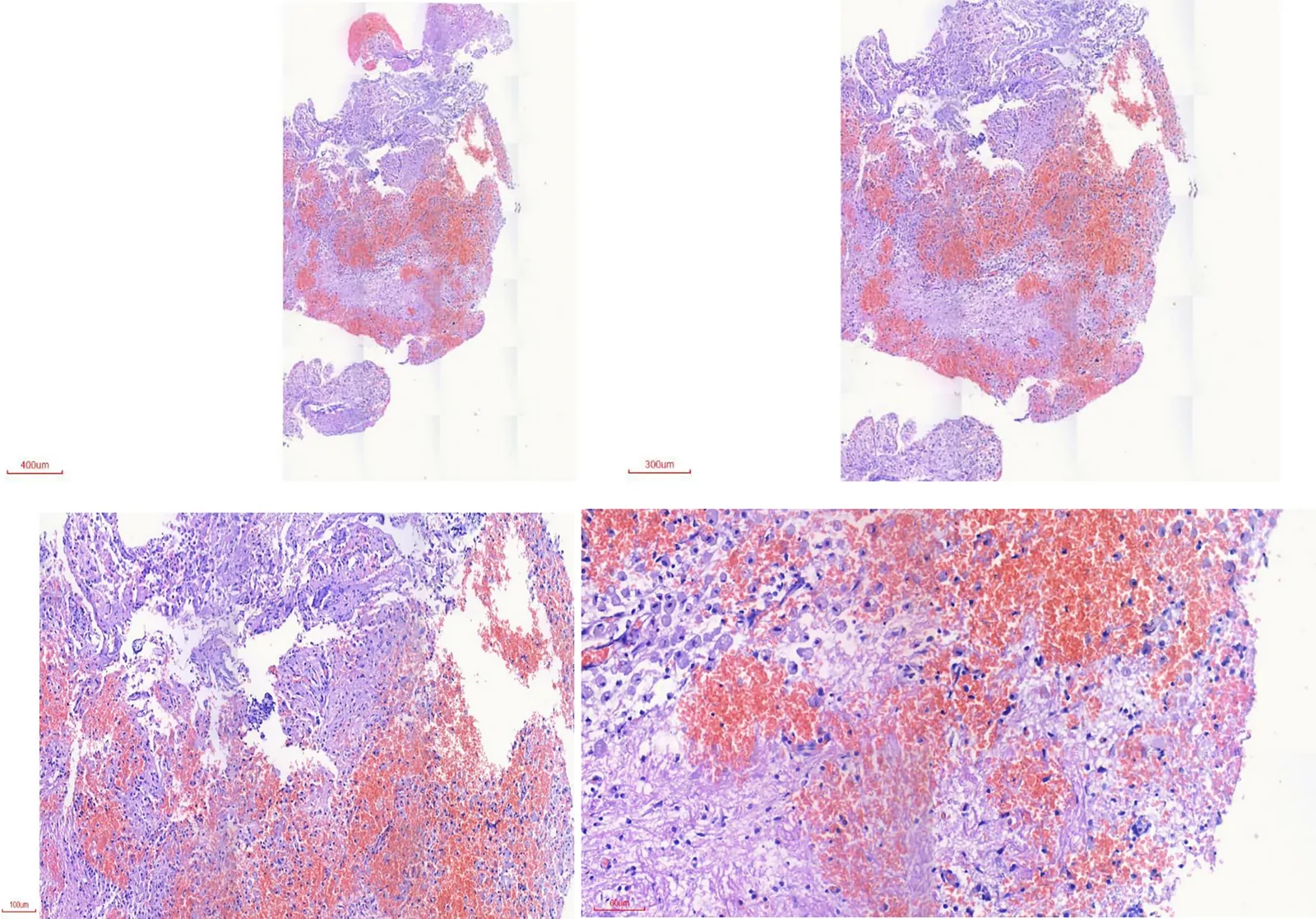
Microscopic section showing neural tissue with reactive glial cells showing ballooning and cytoplasmic clearing. There are edema, hemorrhage, and capillary proliferation with no atypical cells seen

Histology report of the biopsied tissue specimen made a pathologic diagnosis of an inflammatory lesion with no evidence of malignancy (Fig. 4).

She had a tracheostomy sited on postoperative day 12 and was maintained on continuous positive airway pressure ventilatory mode afterward. She developed sudden-onset respiratory distress on postoperative day 27 with consequent hemodynamic instability. Efforts at cardiopulmonary resuscitation were not successful as she died some minutes later.

## Discussion

Neuromyelitis optica spectrum disorder is an autoimmune disease condition in which antibodies are elaborated against aquaporin-4 IgG domiciled in the astrocytic end foot process initiating a cascade of cell-mediated antibody and complement-dependent destruction of astrocytes leading to loss of oligodendrocytes and extensive demyelination in the central nervous system [2, 7].

It is predominantly a disease with female preponderance affecting middle-aged women [2], of which the patient fits the profile.

The pathogenesis of the condition involves risk factors such as infection, oxidative stress and immune tolerance dysfunction leading to local inflammation, pathogenic antibody formation, and denudation of the blood–brain barrier [7]. Our patient had a poorly treated upper respiratory tract infection a month before the onset of symptoms which could have served as a predisposing risk factor. She also showed raised serum titers of acute phase proteins such as C-reactive protein and D-dimer which are markers of inflammation.

She manifested key cardinal diagnostic clinical features of the disease such as area postrema syndrome, transverse myelitis, and brainstem syndromes [6] manifesting as difficulty with swallowing and breathing and limb weakness [8], as well as brain and cervical MRI features in keeping with a possible right optic neuritis and longitudinally extensive transverse myelitis, respectively. Other MRI diagnostic requirements which were not apparent in our patient include area postrema/dorsal medulla lesions, and periependymal brainstem lesions [6].

Her histopathologic slides showed reactive glial cells with cytoplasmic clearing, edema and capillary proliferation in keeping with the neuroinflammatory process. This is similar to findings of axonal damage, eosinophilic infiltration, microglia cell activation, and perivascular immunoglobulin deposition typically seen in Devic’s disease [9].

Given the relative rarity of the condition in our environment and the clinical presentation of the patient, our initial diagnosis was a possible foramen magnum lesion. The cervical MRI finding made us consider an intramedullary spinal cord lesion. The rapidly progressive nature of the course of her symptoms informed the decision for ICU admission, mechanical ventilation, and surgical decompression of the spine to relieve symptoms and obtain biopsy tissue for histologic analysis. We also entertained other possible medical differential diagnoses such as myelin oligodendrocyte disorder, neuromyelitis optica spectrum disorder, and multiple sclerosis, which motivated the need to request serum aquaporin-4 antibodies and oligoclonal bands to confirm diagnosis and rule out other differentials. However, due to the rare presentation of neuromyelitis optica spectrum disorder in our hospital and consequent unavailability of laboratory reagents to assay for aquaporin-4 IgG antibodies, her blood sample were sent to another facility for analyses of which the result returned affirmative on the 2nd day after the patient had surgical cervical spine decompression.

Though there has been documented evidence of marked postoperative neurologic improvement in 89% of 19 patients with compressive cervical myelopathy due to CNS demyelinating disease [10], with another case report of incidental intraoperative finding of neuromyelitis optica spectrum disorder in a patient who had symptomatic relief following surgical decompression and instrumental fusion having presented with radiologic features in keeping with ossified posterior longitudinal ligament of the cervical and thoracic spine [11], it is pertinent to note that early diagnosis and prompt medical treatment are hallmarks in ameliorating morbidity and mortality from the disease process [10].

Our patient had a cell-based assay of serum AQP4 in the course of her evaluation which is the recommended standard of laboratory investigative modality with a sensitivity and specificity yield of 80–100% and 86–100%, respectively [6, 8]. This has an advantage over enzyme-linked immunosorbent assay (ELISA) test with a higher yield of false-positive results [8]. However, it has been noted that the serum levels of AQP4 IgG do not reliably predict the course of the disease nor response to therapy, thus creating a need for biomarkers that could gauge the course and severity of the disease [12].

The aim of immunosuppressive therapy includes preventing relapses and increasing the chances of recuperation from acute disease exacerbation while attenuating the risk of disability [2]. Medications that have been used in the treatment and control of disease progression include mycophenolate mofetil, rituximab, azathioprine, and prednisolone [13, 14]; she had the latter two among other medications and showed a reduction of respiratory distress following commencement of therapy.

Elucidation of the immunopathogenesis of the disease has led to the discovery of monoclonal antibodies that modify complement system activation, decrease antibody production and block IL-6 receptor; this has been reflected in the mechanism of action of novel therapies such as eculizumab, inebilizumab, and satralizumab, respectively [13, 14]. Immune reconstitution treatments such as autologous transplant of stem cell bone marrow have demonstrated some clinical improvement in disease course, reducing progression to disability, and inducing remission of clinical symptoms and radiologic features of disease [15, 16].

Our patient developed sudden-onset respiratory distress, which culminated in her demise. While this could be attributable to a possible relapse, she had some of the risk factors associated with increased propensity for relapse such as younger age, female sex and either African or European descent, having a short disease duration that could have predisposed her to more episodes of acute exacerbation [17, 18]. Relapses could be heralded by tonic spasms and area postrema syndrome with the recovery of two-thirds of cases following early administration of high-dose corticosteroids with or without plasma exchange therapy administered within 2 weeks of the onset of symptoms [19].

## Conclusion

This article highlights an incidence of neuromyelitis optica spectrum disorder and the need to entertain a high index of suspicion of its existence while evaluating potential patients who present with compressive features presumably attributable to cervical spine tumors. It underscores that the hallmark of medical management comprises the use of immunosuppressive therapy, steroids, and plasma exchange therapy. Given its pathophysiologic mechanism of intrinsic neuroinflammation rather than extrinsic mechanical compression, surgical decompression of the spine may not confer a significant benefit in progression-free survival in affected patients.

## Data Availability

The data used in the write-up of this manuscript is available on request from the corresponding author of the manuscript.

## Abbreviations

AQP4: Aquaporin-4
MRC: Medical Research Council
MOG: Myelin oligodendrocyte glycoprotein
CPAP: Continuous positive airway pressure
POD: Postoperative day
MRI: Magnetic resonance imaging
ELISA: Enzyme-linked immunosorbent assay
IgG: Immunoglobulin G
